# *In silico* discovery and serological validation of *Trypanosoma cruzi*-specific B-cell epitopes for high-precision Chagas disease diagnosis

**DOI:** 10.3389/fmicb.2026.1855913

**Published:** 2026-07-13

**Authors:** Mayron Antonio Candia-Puma, Luis Daniel Goyzueta-Mamani, Haruna Luz Barazorda-Ccahuana, Raquel S. B. Câmara, Isabela A. G. Pereira, Ana L. Silva, Maíza M. Rodrigues, Bárbara P. N. Assis, Ana T. Chaves, Laís A. V. A. Corrêa, Manoel O. da Costa Rocha, Denise U. Gonçalves, Ana Alice Maia Gonçalves, Airam Barbosa de Moura, Alexsandro Sobreira Galdino, Ricardo Andrez Machado-de-Avila, Rodolfo Cordeiro Giunchetti, Eduardo Antonio Ferraz Coelho, Miguel Angel Chávez-Fumagalli

**Affiliations:** 1 Computational Biology and Chemistry Research Group, Vicerrectorado de Investigación, Universidad Católica de Santa María, Arequipa, Peru; 2 Escuela de Postgrado, Universidad Católica de Santa María, Arequipa, Peru; 3 Programa de Pós-Graduação em Ciências da Saúde: Infectologia e Medicina Tropical, Faculdade de Medicina, Universidade Federal de Minas Gerais, Belo Horizonte, Brazil; 4 Hospital Eduardo de Menezes, Fundação Hospitalar do Estado de Minas Gerais, Belo Horizonte, Brazil; 5 Microorganism Biotechnology Laboratory, National Institute of Science and Technology on Industrial Biotechnology (INCT-BI), Federal University of São João Del-Rei, São João Del-Rei, Brazil; 6 Laboratório de Fisiopatologia Experimental, Programa de Pós-Graduação em Ciências da Saúde, Universidade do Extremo Sul Catarinense, Santa Catarina, Brazil; 7 Instituto Nacional de Ciência e Tecnologia em Biotecnologia Industrial (INCT-BI), Distrito Federal, Brasilia, Brazil; 8 Facultad de Medicina, Universidad de Rio Verde, Fazenda Fontes do Sabre, Campus Universitário, Rio Verde, Goiás, Brazil; 9 Laboratório de Biologia das Interações Celulares, Instituto de Ciências Biológicas, Universidade Federal de Minas Gerais, Belo Horizonte, Brazil; 10 Instituto Nacional de Ciência e Tecnologia em Doencas Tropicais (INCT-DT), Salvador, Brazil

**Keywords:** B-cell epitopes, Chagas disease, diagnostics, *in silico*, *Trypanosoma cruzi*

## Abstract

Chagas disease is caused by the parasite *Trypanosoma cruzi* and remains a neglected tropical disease presenting a substantial global health burden. Crude antigen-based assays have historically been limited in specificity; however, even contemporary recombinant-antigen tests may exhibit residual cross-reactivity, depending on antigen composition and geographic context. To overcome this limitation, this study developed a novel diagnostic strategy that integrates computational and experimental approaches to identify specific linear B-cell epitopes within the *T. cruzi* proteome. The strategy was developed to exclude sequences homologous to *H. sapiens* and *Leishmania* spp. proteins, thereby minimizing potential cross-reactivity. Using a consensus approach across five prediction algorithms, B-cell epitopes were identified and subsequently clustered to reveal conserved, immunoreactive consensus sequences. The peptide sequences were characterized for optimal physicochemical properties and subsequently modeled to interact with a human antibody using protein-peptide docking and molecular dynamics simulations to assess complex stability. The most promising candidates were chemically synthesized and validated using ELISA against a cohort comprising Chagas disease patients (chronic indeterminate and cardiac forms), healthy donors, and a cross-reactive control group (visceral and tegumentary leishmaniasis and leprosy). From the initial set of 19,245 proteins, the multi-tiered bioinformatic analysis identified 4,431 unique, non-homologous sequences. Consensus prediction yielded 401 high-confidence epitopes, which were refined to 179 structurally stable candidates. Computational analyses identified five top-ranking epitopes capable of forming high-affinity, stable complexes with a human antibody. Experimental validation confirmed the high diagnostic accuracy of two epitopes, which demonstrated exceptional diagnostic performance: Epitope 4 and Epitope 5 achieved 100% sensitivity within the evaluated cohort. Notably, Epitope 5 exhibited superior specificity, reaching 96.67% against healthy controls and 90.91% against the cross-reactive group. This study establishes a basis for the development of an improved immunoassay for Chagas disease and provides a reproducible framework for targeted epitope discovery. Consequently, this study validates a high-precision computational pipeline capable of discovering *T. cruzi*-specific antigens that effectively circumvent cross-reactivity with *Leishmania* spp., proposing Epitope 5 as a qualified candidate for reliable serological diagnosis in co-endemic regions.

## 1 Introduction

Chagas disease (CD), caused by the hemoflagellate parasite *Trypanosoma cruzi*, constitutes a neglected tropical disease of global impact with devastating consequences (OMS, 2025). Classified by the WHO as one of the deadliest parasitosis in the Americas, it affects an estimated 6–7 million people in endemic regions of Latin America, with 12,000 deaths each year, mainly from Chagasic cardiomyopathy, megacolon, and sudden death (Lidani et al., 2019). Globalization has extended its presence to more than 30 non-endemic countries through migration and blood transfusion, transforming it into a transcontinental challenge (Requena-Méndez et al., 2014). This epidemiological burden underscores the urgency of precise diagnostics, particularly during indeterminate chronic phases and in blood banks, where serological testing is indispensable.

In the complex landscape of CD, where the often-silent progression of pathology and low parasitemia render direct parasitological methods insufficient during the chronic stage, serology stands as the definitive bridge to timely patient management and epidemiological control (Schijman et al., 2024). Consequently, the detection of specific antibodies is the cornerstone of CD diagnosis. The distinct roles of immunoglobulin isotypes (IgG, IgM, and IgA) provide critical insights into the stage and dynamics of the infection. IgM antibodies are the first responders, appearing during the acute phase, but their transient nature and lower titers make them less reliable for routine screening (Candia-Puma et al., 2022; Peverengo et al., 2025). In contrast, IgG antibodies emerge later and persist indefinitely throughout the chronic phase, making them the primary target for confirmatory serological testing in individuals with suspected chronic infection and for blood bank screening (Brossas et al., 2021). Notably, the detection of IgA, though less routinely employed, has shown promise as a potential marker of active infection and therapeutic response, as its levels may decline more rapidly after successful treatment compared to total IgG (Morgan et al., 1998; Pinazo et al., 2014). Therefore, a comprehensive diagnostic tool should ideally account for these isotypes to maximize clinical utility across different phases of the disease.

Nevertheless, the current diagnostic standard, two serological assays (ELISA, HAI, and IIF) employing crude (Gomes et al., 2009) or semi-purified (Oelemann et al., 1998) antigens, suffers critical limitations that compromise reliability. Most notably, low specificity arises from cross-reactivity with *Leishmania* spp. (especially *L. braziliensis* and *L. infantum*), syphilis and autoimmune diseases, yielding up to 15% false-positive results in co-endemic areas (Candia-Puma et al., 2022; Schijman et al., 2024). Additional issues include reduced sensitivity in immunocompromised individuals, lack of standardization among commercial kits, and failure to discriminate active from resolved infection (Candia-Puma et al., 2022; Otani et al., 2009; Sánchez-Camargo et al., 2014). These shortcomings carry serious clinical (erroneous treatment, patient anxiety) and operational (discarded blood units, elevated costs) consequences, highlighting the pressing need for high-precision diagnostic alternatives.

In this context, chemically synthesized soluble linear B-cell epitopes emerge as a promising solution. These short peptides (< 20 amino acids), corresponding to specific antigenic regions recognized by antibodies, enable the exclusion of non-specific immunogenic components present in crude extracts, the principal source of cross-reactivity (Zhou et al., 2022). Their chemical synthesis facilitates the development of highly standardized, reproducible, and scalable assays, with the potential to rationally optimize both sensitivity and specificity (Caoili, 2022; Ribeiro et al., 2024). Crucially, synthetic peptides are inherently stable and do not require a cold chain for storage and transport (Gomara and Haro, 2007; Pandey et al., 2021). This attribute represents a significant logistical advantage for the deployment of diagnostic tests in remote, rural areas (where Chagas disease is predominantly endemic) and where infrastructure, including reliable electricity for refrigeration, is often limited or unavailable (Perez et al., 2024).

Traditional epitope identification *via* immunological screening, however, is inherently slow, costly, and low-throughput (Grifoni et al., 2020). To circumvent these limitations, *in silico* strategies offer a robust alternative. Bioinformatic tools (such as BepiPred, ABCPred, and APP) facilitate the rapid and cost-effective prediction of candidate epitopes directly from protein sequences (Sun et al., 2019; Zheng et al., 2023). Yet, while previous studies have applied these computational approaches to Chagas disease (Gállego et al., 2020; Mucci et al., 2017), a critical gap remains: the identification of epitopes with exclusive specificity for *T. cruzi*. Prior efforts have struggled to effectively filter out conserved antigenic regions shared with *Leishmania* spp. and other phylogenetically related pathogens, specifically *Trypanosoma rangeli*, leading to persistent cross-reactivity (Schijman et al., 2024). This limitation is particularly detrimental in co-endemic regions of South America, where the high degree of sequence homology between these parasites makes serological discrimination between Chagas disease and leishmaniasis a formidable challenge.

This study proposes an integrated *in silico*, experimental approach to identify and validate a *T. cruzi*-specific linear B-cell epitope. The central hypothesis suggests that rigorous bioinformatic design, focused on excluding homology with the proteomes of *Leishmania* spp. and *Homo sapiens*, can yield peptides that, when implemented in ELISA, achieve high sensitivity and specificity, thereby eliminating false positive results. By closing this critical gap, the study aims to provide a transformative diagnostic tool for epidemiological control. Therefore, this study aimed to identify and experimentally validate species-specific linear B-cell epitopes from *T. cruzi* for diagnostic application.

## 2 Material and methods

### 2.1 *In silico* prediction of *T. cruzi*-specific B-cell epitopes

#### 2.1.1 Obtaining and initial processing of the reference proteome

The reference proteome of *T. cruzi* was obtained from the UniProt database (http://www.uniprot.org; Bateman et al., 2025), prioritizing a high-coverage, non-redundant protein set. This specific strain was selected as the reference because it serves as the widely accepted gold standard in *T. cruzi* genomics, providing the highest quality of genomic assembly and annotation currently available. Although a single strain does not encompass the full spectrum of global genetic diversity, it provides the most robust and reliable bioinformatic foundation for an initial, unbiased proteome-wide screening. Initial physicochemical characterization—including molecular weight (kDa) and isoelectric point (pI)—was performed for all proteins using the Compute pI/Mw tool on the ExPASy server (https://web.expasy.org/compute_pi/; Gasteiger et al., 2005). At this stage, manually reviewed proteins (curated entries) were deliberately excluded. This exclusion was a strategic decision, as reviewed proteins typically harbor classical immunodominant antigens that are the primary source of the pan-trypanosomatid cross-reactivity this study aims to circumvent. Therefore, focusing the analysis exclusively on the uncharacterized (unreviewed) proteome ensures the discovery of entirely novel diagnostic markers.

#### 2.1.2 Filtering by homology with host and related parasites

To diminish potential cross-reactivity in diagnostic applications, sequences homologous to *H. sapiens* (taxid:9606) and the entire *Leishmania* genus (taxid:5658, which computationally encompasses all sequenced *Leishmania* species) were excluded. Other phylogenetically related pathogens were not included in this initial *in silico* filtering step to focus exclusively on the primary co-endemic interference. Each protein in the reference proteome underwent pairwise comparison against both host and parasite databases using BLASTp (https://blast.ncbi.nlm.nih.gov/Blast.cgi; Altschul et al., 1990). Homology was defined by stringent thresholds: an e-value ≤ 0.005, a bit score ≥100.0, and/or a sequence identity ≥ 30%. Consequently, sequences showing homology were excluded, while those lacking significant similarity were retained for downstream analyses to ensure candidate epitopes are uniquely recognizable as *T. cruzi*-specific antigens.

#### 2.1.3 Prediction of linear B cell epitopes

Linear B-cell epitope prediction was performed using five servers: AAP (http://ailab-projects2.ist.psu.edu/bcpred/predict.html; Chen et al., 2007; Pandey et al., 2020), ABCpred (https://webs.iiitd.edu.in/raghava/abcpred/ABC_submission.html; Chen et al., 2020; Saha and Raghava, 2006, 2007), BCpred (http://ailab-projects2.ist.psu.edu/bcpred/predict.html; Chen et al., 2020; El-Manzalawy et al., 2008a,b; Saha and Raghava, 2006), BepiPred-2.0 (https://services.healthtech.dtu.dk/services/BepiPred-2.0/; Dai et al., 2020; Jespersen et al., 2017, 2019), and FBCPred (http://ailab-projects2.ist.psu.edu/bcpred/predict.html; Chen et al., 2020; El-Manzalawy et al., 2008a,b; Saha and Raghava, 2006). These servers incorporate distinct computational algorithms—support vector machines, recurrent neural networks, string kernel methods, random forests, and subsequence kernel methods, respectively—to identify epitopes with a high likelihood of efficient processing by B cells. A specific prediction score threshold was applied for each algorithm, selected to optimize specificity and ensure high-confidence selections. The exact threshold values used are detailed in Table 1.

**Table 1:** Linear predictors of B-cell epitopes.

Tool	Method	Prediction threshold	Specificity	Epitope size	Web link	References
AAP	Support vector machine + amino acid pair antigenicity scale	0.70	75%	12	http://ailab-projects2.ist.psu.edu/bcpred/predict.html	Chen et al., 2007; Pandey et al., 2020
BCpred	Support vector machine + k-mer subsequence kernel	0.80	75%	12	http://ailab-projects2.ist.psu.edu/bcpred/predict.html	Chen et al., 2020; El-Manzalawy et al., 2008a,b; Saha and Raghava, 2006
FBCPred	Support vector machine + flexible subsequence kernel	0.85	75%	12	http://ailab-projects2.ist.psu.edu/bcpred/predict.html	Chen et al., 2020; El-Manzalawy et al., 2008a,b; Saha and Raghava, 2006
ABCpred	Recurrent neural network (Jordan network)	0.80	95.50%	12	https://webs.iiitd.edu.in/raghava/abcpred/ABC_submission.html	Chen et al., 2020; Saha and Raghava, 2006, 2007
BepiPred-2.0	Random forest + physicochemical features	0.55	81.66%	≥ 12	https://services.healthtech.dtu.dk/services/BepiPred-2.0/	Dai et al., 2020; Jespersen et al., 2017, 2019

#### 2.1.4 Identification of consensus epitopes

To identify candidate epitopes with a higher likelihood of being immunoreactive and thus prioritize them for the development of a diagnostic test, the resulting epitopes were compared, and consensus epitopes were determined using the clustering tool for similar sequences from the Immune Epitope Database (IEDB; http://tools.iedb.org/cluster/; Dhanda et al., 2018). This consensus approach is based on its ability to rank candidates according to their conservation and likelihood of immune response. The parameters used included a minimum sequence identity threshold of 70% and a peptide length ranging from 12 to 16 amino acids (Fonseca et al., 2025; Lu et al., 2024).

#### 2.1.5 Analysis of physicochemical properties

The physicochemical properties of the selected sequences were analyzed using the ProtParam tool on the ExPASy server (https://web.expasy.org/protparam/; Gasteiger et al., 2005) to determine the following parameters: (1) amino acid composition; (2) theoretical pI calculated using the Bjellqvist method; (3) stability predictors, including the instability index (values >40 indicating instability) and aliphatic index (thermostability proxy); and (4) hydrophobicity *via* the grand average of hydropathicity (GRAVY; positive values denote hydrophobic character).

#### 2.1.6 Retrieval of structural, sequential data and delineation of the paratope

A search was conducted in the Protein Data Bank (PDB; https://www.rcsb.org/) to identify three-dimensional entries of human immunoglobulins from the IgA (3M8O; Correa et al., 2013), IgG (1MIM; Mikol, 1996), and IgM (1DN0; Cauerhff et al., 2000) classes. For each structure, the.pdb files and FASTA sequences of the heavy and light chains were downloaded simultaneously, ensuring that primary and structural data were linked from the outset of the analysis. If an entry contained missing regions or incomplete domains, these portions were reconstructed through homology modeling using the SWISS-MODEL server (https://swissmodel.expasy.org/; Waterhouse et al., 2018). For reconstructing human Fab fragments where only small loops are missing and there is near-perfect sequence identity with templates resolved by crystallography, this template-based homology approach is precise and robust, making it a highly suitable choice over *de novo* AI-based folding algorithms. This process generated complete models with appropriate confidence levels. The stereochemical quality and backbone conformation integrity of all initial templates and the subsequently generated homology models were rigorously validated. This was achieved by analyzing the phi (ϕ) and psi (ψ) dihedral angle distributions *via* Ramachandran plots computed using the PROCHECK software suite (v3.5.4; Laskowski et al., 1993). Given the presence of flexible linker regions and loops characteristic of immunoglobulin domains, a model was considered acceptable for subsequent analysis if a minimum of 90% of all non-glycine, non-proline residues were located within the most favored and additionally allowed regions of the conformational space (Hooft et al., 1997). All refined models met or exceeded this threshold, ensuring the structural plausibility of the rebuilt domains for our comparative analysis. The heavy and light chains were analyzed in parallel using IMGT/DomainGapAlign (https://www.imgt.org/3Dstructure-DB/cgi/DomainGapAlign.cgi, accessed on 04 October 2024), Parapred (https://www-cohsoftware.ch.cam.ac.uk/index.php/Parapred, accessed on 04 October 2024), and LYRA (http://tools.iedb.org/lyra/, accessed on 04 October 2024), three complementary algorithms for predicting antibody-antigen contact regions (Ehrenmann et al., 2010; Klausen et al., 2015; Liberis et al., 2018). Overlapping the results enabled the construction of a consensus paratope sequence, minimizing tool-specific biases and enhancing the reliability of identifying critical residues for antigen recognition.

#### 2.1.7 Epitope modeling

Epitopes deemed stable in the previous stage underwent *de novo* three-dimensional prediction using the PEP-FOLD 3.5 server (https://mobyle.rpbs.univ-paris-diderot.fr/cgi-bin/portal.py#forms::PEP-FOLD3, accessed on 09 October 2024; Lamiable et al., 2016). For each linear sequence, conformational sampling was performed utilizing the coarse-grained sOPEP (single-point optimized potential for efficient peptide structure prediction) force field, with the default parameters recommended for peptides spanning 5–50 residues. The primary quantitative metric for evaluating the energetic quality and conformational stability of each generated model was the sOPEP Energy. This score serves as the key thermodynamic indicator of modeling efficiency within the PEP-FOLD framework, where a lower, more negative value correlates with a more stable, energetically favorable conformation, reflecting a higher likelihood of representing a native-like fold in solution (Binette et al., 2022).

#### 2.1.8 Protein-peptide molecular docking and molecular dynamics

Selected Fab fragments and epitopes were docked with HPEPDOCK 2.0 (http://huanglab.phys.hust.edu.cn/hpepdock) and HawkDock (http://cadd.zju.edu.cn/hawkdock/), engines integrating peptide flexibility and free energy estimation (Weng et al., 2019; Zhou et al., 2018). Top-scoring complexes were visually inspected in UCSF ChimeraX (v1.11) to validate binding geometries consistent with the paratope and rule out computational artifacts. To prioritize computational resources, only complexes with a docking score exceeding the mean plus one standard deviation, calculated independently for each software's specific scoring distribution, retained (a statistical outlier threshold that, under a normal distribution, targets the upper ~16% of results from each independent screen; Jideani et al., 2024). This threshold balanced sensitivity and specificity, focusing simulations on interactions with a higher likelihood of biological relevance.

Molecular dynamics (MD) simulations of the antibody-epitope complexes were performed using the GROMACS 2026.2 software package. The systems were parameterized using the CHARMM27 all-atom force field. Each complex was centered in a cubic simulation box, ensuring a minimum distance of 1.2 nm between the protein complex and the box boundaries, and subsequently solvated using the TIP3P explicit water model. To achieve electrostatic neutrality, sodium (Na+) and chloride (Cl-) ions were added to the solvated systems. Before the production dynamics, the systems were subjected to energy minimization to eliminate unfavorable steric clashes and optimize system geometry. Following minimization, the systems underwent a two-stage equilibration process consisting of an NVT (constant number of particles, volume, and temperature) ensemble equilibration to stabilize the temperature, followed by an NPT (constant number of particles, pressure, and temperature) ensemble equilibration to stabilize system density and pressure.

The production MD simulations were carried out for 500 ns for each system in independent triplicates, employing GPU acceleration for the calculation of non-bonded interactions and Particle Mesh Ewald (PME) electrostatics. Post-simulation, the raw trajectories were processed to remove periodic boundary condition (PBC) artifacts; molecules were made whole, centered using the receptor as the reference, and subjected to rotational and translational fitting to eliminate rigid-body motions. All downstream biophysical analyses were performed on these aligned trajectories using built-in GROMACS modules. Structural stability and system compactness were evaluated by computing the Root Mean Square Deviation (RMSD) of the protein backbone and the Radius of Gyration (RG) using the gmx rms and gmx gyrate tools, respectively. Thermal mobility and local flexibility were assessed by calculating the Root Mean Square Fluctuation (RMSF) per atom (gmx rmsf), while the degree of solvation was determined by calculating the Solvent Accessible Surface Area (SASA) utilizing the gmx sasa module. Finally, the atomic contacts defining the antibody-epitope interaction interfaces were quantified using the gmx mindist module, applying a strict distance cutoff of < 0.4 nm.

### 2.2 Serological evaluation of candidate epitopes against sera from patients

#### 2.2.1 Chemical synthesis of peptides

Candidate epitopes, selected based on *in silico* screening for immunogenicity and specificity, were synthesized using standard Fmoc (9-fluorenylmethoxycarbonyl) solid-phase peptide synthesis (SPPS) on a polystyrene resin, following previously described protocols (Borghezan et al., 2025; Câmara et al., 2024b; Machado de Avila et al., 2011; Merrifield, 1969). The process was meticulously executed through iterative cycles of N-terminal Fmoc deprotection with 25% (v/v) 4-methyl-piperidine in dimethylformamide (DMF), followed by extensive washing with DMF and dichloromethane to remove reaction byproducts. Subsequent coupling of each Fmoc-protected amino acid was qualitatively verified after each cycle using the bromophenol blue test to ensure reaction completion. Following the assembly of the full sequence, the nascent peptide was simultaneously cleaved from the resin and globally deprotected *via* treatment with a chilled cleavage cocktail—a precise mixture of trifluoroacetic acid (TFA; 95% v/v), triethylsilane (TES; 2.5% v/v) as a carbocation scavenger, and ultrapure water (2.5% v/v)—for 3 h under gentle agitation. The crude peptide was then precipitated in a copious volume of cold diethyl ether and incubated at −20 C for 18 h to maximize yield. The resulting pellet was isolated by centrifugation at 4500 × g for 10 min at 4 C, subjected to repeated ether washes to purge organic impurities, and finally lyophilized to obtain the pure product as a stable powder, which was stored at −20 C and reconstituted in Milli-Q water at 1.0 mg/mL immediately before use. Five peptides were synthesized and named epitope 1–5.

#### 2.2.2 Ethical statement and study population

The study was approved by the Ethics Committee of the Federal University of Minas Gerais (UFMG, Belo Horizonte, Minas Gerais, Brazil), logged under protocol number CAAE-32343114.9.0000.5149. All participants were adults and signed the informed consent form at the moment of inclusion.

The study followed a single-center, case-control, phase-I diagnostic accuracy design using controlled archival samples (Zapf et al., 2020). For this, a total of 178 subjects were enrolled in the study. Blood was obtained through venipuncture using a sterile 20 mL vacuum collection tube with clot activator and separator gel. Subsequently, tubes were centrifuged at 3,500 x *g* for 15 min at 4 C, and the serum was separated and stored at −70 C until use. A total of 88 serum samples were obtained from Chagas disease patients from the Center for Training and Reference in Infectious and Parasitic Diseases (CTR DIP-Orestes Diniz) in Belo Horizonte, Minas Gerais, Brazil. Patients are presented with an indeterminate form (*n* = 43) or Chagas cardiomyopathy (*n* = 45) clinical disease. Parasitological confirmation of *T. cruzi* infection was performed by hemoculture in combination with specific ELISA or indirect hemagglutination assay (IHA). Leishmaniasis was diagnosed by clinical evaluation and parasitological test for tegumentary leishmaniasis (TL; Giemsa-stained smears from lesion fragments and PCR to detect *L. braziliensis* kDNA) and visceral leishmaniasis (VL; aspirates from spleen and/or bone marrow and PCR to detect *L. infantum* kDNA; *n* = 20). TL was clinically stratified in cutaneous (*n* = 10) and mucosal (*n* = 10) leishmaniasis. Leprosy was diagnosed by clinical evaluation, slit-skin smears, and histopathological examination of the lesion biopsy. Patients were classified according to WHO criteria in presenting either multibacillary (MB, *n* = 10) or paucibacillary (PB, *n* = 10) form. Sera samples from healthy subjects living in the endemic region of disease, Poços de Caldas, in the state of Minas Gerais (*n* = 30), who did not present clinical signs or symptoms of any infectious disease at the moment of collection, were also used.

#### 2.2.3 ELISA

Although the *in silico* pipeline modeled interactions with IgA, IgM, and IgG to explore broad pan-isotypic recognition potential, the experimental serological validation was strictly focused on IgG. This strategic decision is grounded in clinical diagnostic standards: persistently elevated anti-*T. cruzi* IgG titers constitute the definitive gold standard biomarker for chronic Chagas disease, which corresponds directly to the clinical status of the patient cohort evaluated in this study.

Previous titration curves were performed to determine the most appropriate concentration of antigens and antibody/sample dilutions to be used in the ELISA experiments. Briefly, 96-well high-binding plates (Thermo Fisher, USA) were coated with each synthetic peptide (4, 6, 4, and 8 μg per well, respectively), which were diluted in carbonate buffer pH 9.6 and incubated for 1 h at 37 C. Wells were blocked with 200 μL of Phosphate Buffered Saline (PBS 1x pH 7.4) plus Tween 20 (PBS-T) containing 2% (w/s) casein for 1 h at 37 C. Plates were washed three times with PBS-T and incubated with 50 μL/well of serum samples, which were diluted 1:100 in PBS-T containing 2% casein, for 1 h at 37 C. After washing plates three times with PBS-T, 100 μL/well of peroxidase-conjugated anti-human IgG antibody (A1811, Invitrogen, USA), which was diluted 1:5,000 in PBB-T plus 2% casein, was added to the wells, and plates were incubated for 1 h at 37 C. Next, wells were washed three times with PBS-T, and reactions were developed by adding 3,3′,55′; tetramethylbenzidine (TMB, Scienco, Brazil) at room temperature for 20 min and in the dark. Reactions were stopped by adding 50 μL of 2N H_2_SO_4_, and the optical density (OD) values were read in a microplate spectrophotometer (Molecular Devices, Spectra Max Plus, Canada), at 450 nm (Câmara et al., 2024a).

### 2.3 Statistical analysis

Results were entered into Microsoft Excel (version 10.0) spreadsheets and analyzed with GraphPad Prism version 10.0.2 for Windows (GraphPad Software, USA, www.graphpad.com). The cut-off values were calculated through Receiver Operating Characteristic (ROC) curves. The ROC curves were plotted with the values from samples from *T. cruzi*-infected patients vs. those from control groups. For cutoff selection, we use the highest positive likelihood ratio (LR+), defined as the ratio of the probability of a positive test in patients to that in controls. Tables of contingency and Fisher's exact test (*P* < 0.05) were used to calculate: Sensitivity (Se), Specificity (Sp), and the 95 % Confidence Interval (95 % CI). The Kolmogorov-Smirnov normality test was applied, while the Kruskal-Wallis test was followed by Dunn's correction for multiple comparisons was used for group comparisons. Differences were considered significant with *P* < 0.05.

## 3 Results

### 3.1 Comprehensive rational design pipeline for epitope discovery

**Figure 1:**
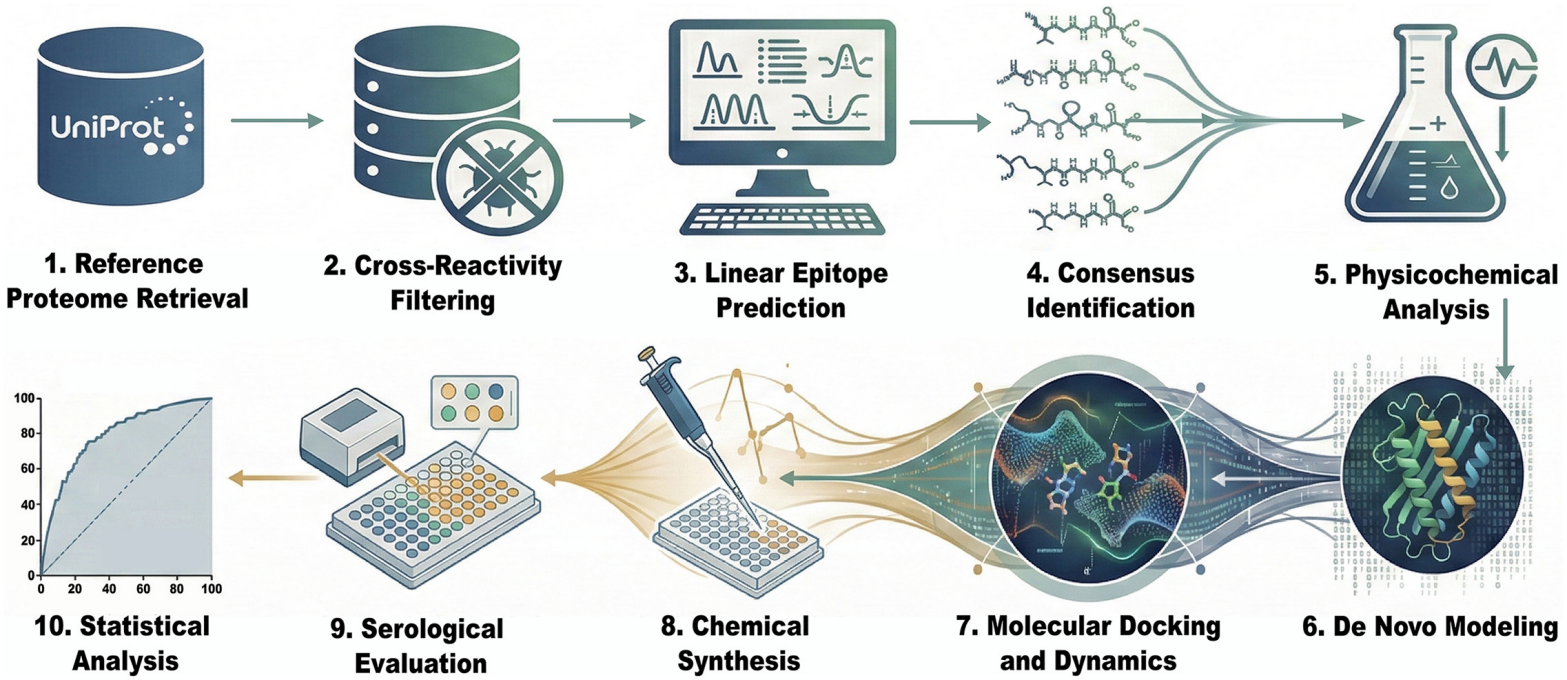
Integrative immunoinformatics and experimental pipeline for the discovery and validation of *T*. cruzi B-cell epitopes.

To systematically identify and validate novel diagnostic markers for Chagas disease, we developed a sequential, ten-step computational and experimental framework, as illustrated in Figure 1. The pipeline initiates with Reference Proteome Retrieval from UniProt (Step 1), followed by rigorous Cross-Reactivity Filtering (Step 2) to eliminate homologous sequences. Candidate sequences are then subjected to Linear Epitope Prediction (Step 3) using multiple algorithms, culminating in a Consensus Identification (Step 4) to minimize false positives. The selected epitopes undergo Physicochemical Analysis (Step 5) to ensure sequence-based physicochemical stability. Promising candidates advance to *De Novo* Modeling (Step 6) and subsequent Molecular Docking and Dynamics (Step 7) to evaluate their theoretical binding affinity and stability with human immunoglobulins. Successfully prioritized targets are then produced *via* Chemical Synthesis (Step 8) and subjected to Serological Evaluation (Step 9) using patient cohorts. Finally, the diagnostic performance of the assays is rigorously evaluated through Statistical Analysis (Step 10).

### 3.2 *In silico* identification of antigenic epitopes

#### 3.2.1 Identification, screening, and physicochemical characterization of non-homologous B-cell epitopes

**Figure 2:**
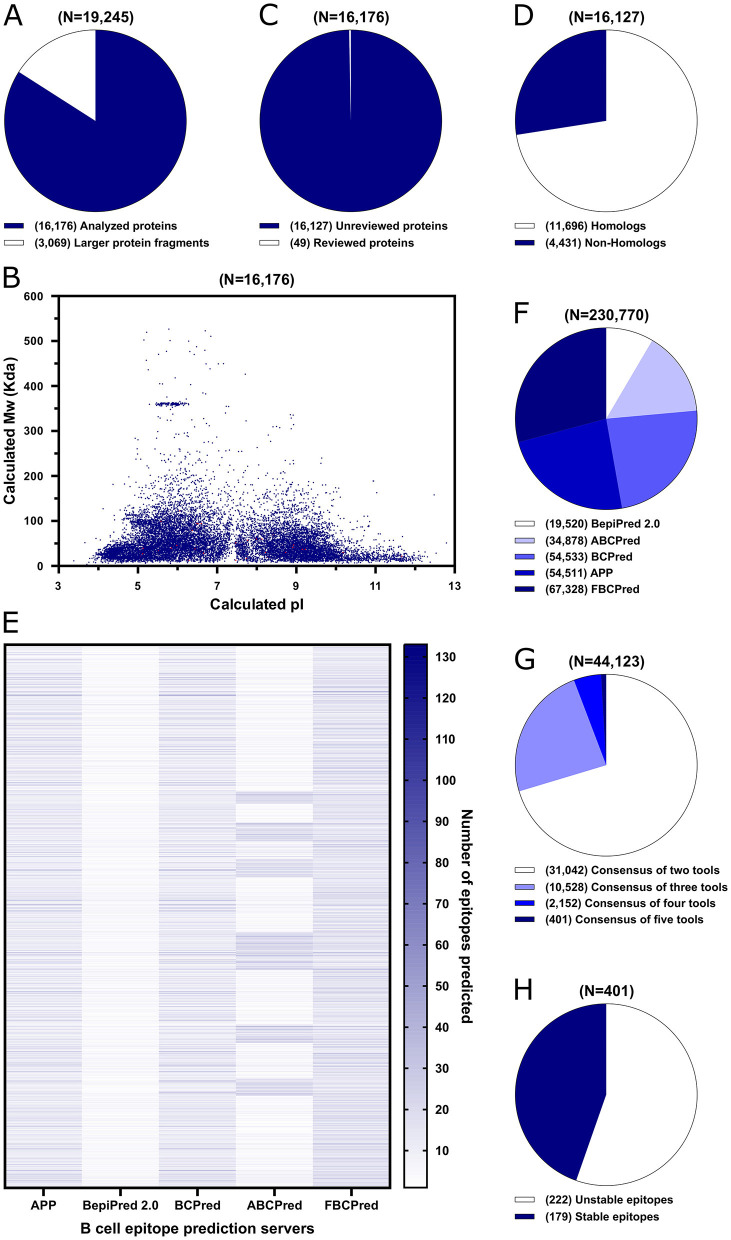
*In silico* analysis of B-cell epitopes in the *T. cruzi* proteome. **(A)** Total proteome (19,245 proteins) excluding truncated fragments (3,069). **(B)** The calculated molecular weight (Mw) and isoelectric point (pI) of 16,176 proteins are provided. The status of the proteins is as follows: unreviewed (blue dots−16,127) and reviewed (red dots−49). **(C)** Selection of unreviewed full-length proteins (16,127) after excluding reviewed ones (49). **(D)** Identification of 4,431 non-homologous proteins (H. sapiens/Leishmania). **(E)** Heatmap of predicted epitopes for non-homologous proteins using five B-cell epitope prediction servers (APP, BepiPred 2.0, BCPred, ABCPred, and FBCPred). **(F)** Prediction of B-cell epitopes in non-homologous proteins using five servers (counts: APP = 54,511, BepiPred = 19,520, BCPred = 54,533, ABCPred = 34,878, FBCPred = 67,328). **(G)** Consensus epitopes: 31,042 (2 tools), 10,528 (3 tools), 2,152 (4 tools), 401 (5 tools). **(H)** 179/401 total consensus epitopes showed structural stability (ProtParam).

The *T. cruzi* reference proteome (strain CL Brener—UniProt ID: 353153) contains 19,245 entries. We applied a quality control filter excluding 3,069 (15.9%) truncated protein fragments, as they impede accurate physicochemical parameter calculation (Figure 2A). To focus our analysis on the uncharacterized proteome, we further excluded the 49 (0.3%) manually reviewed in UniProt (Figure 2B). The final dataset comprises 16,127 unreviewed, full-length proteins (Figure 2C).

Sequence analysis using BLASTp identified 113 sequences with an expected e-value greater than 0.005 and a bit score less than 100.0 against reference proteomes of *H. sapiens* and/or *Leishmania*. Additionally, 4,318 sequences showed no significant similarity. In total, 4,431 sequences (Figure 2D) did not exhibit detectable homology with proteins from these organisms, suggesting the presence of unique or highly specific elements in *T. cruzi*.

To visualize potential epitopes on non-homologous proteins, a heatmap was generated using five specialized B-cell epitope prediction servers: APP, BepiPred 2.0, BCPred, ABCPred, and FBCPred. This diverse strategy, which employs multiple algorithms, enables a comprehensive assessment of regions likely to be recognized by the immune system, accounting for the structural and sequence variability of the target proteins. The heatmap provides an intuitive visual representation of the distribution and intensity of the predicted epitopes (Figure 2E). The total number of epitopes predicted by each server was: APP identified 54,511, BepiPred 2.0 identified 19,520, BCPred identified 54,533, ABCPred identified 34,878, and FBCPred identified 67,328 (Figure 2F). This substantial variability in counts reflects the inherent differences in the algorithms and methods employed by each tool, highlighting how the choice of method significantly influences the identified immunogenic landscape (De Groot et al., 2020; Ogishi and Yotsuyanagi, 2019).

To increase confidence in the predictions, a consensus analysis was performed across the five servers. 31,042 epitopes were identified with agreement across at least two tools. The consensus across three tools yielded 10,528 epitopes, the consensus across four tools yielded 2,152 epitopes, and the highest consensus (five tools) identified 401 epitopes (Figure 2G). This approach stratifies predictions based on the level of agreement, providing a confidence gradient where higher consensus reduces the likelihood of false positives.

Of the 401 epitopes predicted by consensus across the five tools, 179 exhibited stabilities according to the instability index calculated using the ProtParam tool on the ExPASy server (Figure 2H). This predicted physicochemical stability is a crucial attribute for the potential biological functionality of these epitopes, as it increases the likelihood that they will maintain a conformation recognizable by the immune system (Mahboob et al., 2025; Qing et al., 2022).

#### 3.2.2 Structural modeling and validation of epitope-paratope interfaces

Representative human Fabs of each isotype were selected, strictly complying with intact domains, absence of mutations, and human origin, and their linked.pdb files and FASTA sequences were downloaded. The IgA Fab (PDB 3M8O, 1.55 Å) stood out, with R_work 0.153, R_free 0.182, 0% Ramachandran outliers, clash score 9, and only 1.3% side-chain deviations; the IgG Fab (PDB 1MIM, 2.60 Å) showed moderate quality (R_work 0.196, clash score 10, 2.4% outliers, and 12.1% misplaced side chains); and the IgM Fab (PDB 1DN0, 2.28 Å) exhibited similar metrics (R_work 0.180, R_free 0.240, clash score 22, 1.2% outliers, and 12.7% misplaced side chains). Missing regions in any of these entries were completed through homology modeling in SWISS-MODEL, generating comprehensive models with suitable confidence levels before proceeding with further comparative analyses.

**Figure 3:**
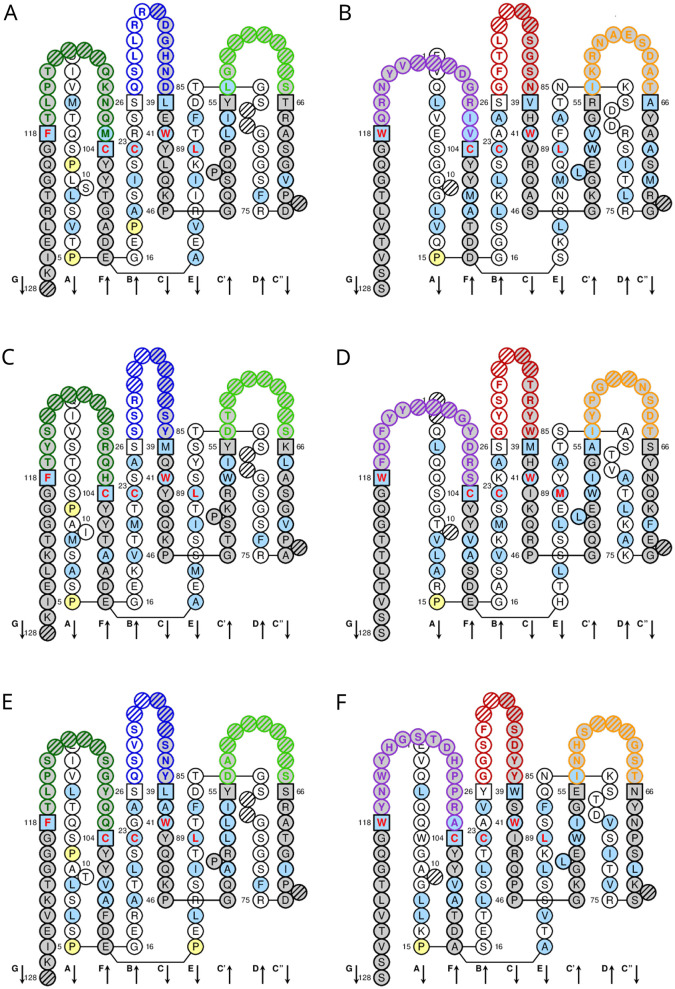
Pearl necklace plot representation of the paratopes in the Fab regions of human immunoglobulins across three isotypes (IgA, IgG, IgM), as determined by the IMGT/DomainGapAlign tool. **(A)** IgA heavy chain, **(B)** IgA light chain, **(C)** IgG heavy chain, **(D)** IgG light chain, **(E)** IgM heavy chain, **(F)** IgM light chain.

The combination of IMGT/DomainGapAlign, Parapred, and LYRA converged on a consensus paratope for each chain, revealing distinct CDR profiles across isotypes: IgA heavy chain (H-CDR1: ASGFTLSGSNV, H-CDR2: RIKRNAESDATA, H-CDR3: VIRGDVYNRQWG; light chain L-CDR1: SCRSSQSLLRRDGHNDLEWY, L-CDR2: IYLGSTRASGV, L-CDR3: YCMQNKQTPLTFG), IgG (H-CDR1: ASGYSFTRYWM, H-CDR2: AIYPGNSDTS, H-CDR3: SRDYGYYFDFWG; L-CDR1: TCSASSSRSYMQWY, L-CDR2: IYDTSKLASGV, L-CDR3: YCHQRSSYTFG), and IgM (H-CDR1: VYGGSFSDYYW, H-CDR2: EINHSGSTN, H-CDR3: ARPPHDTSGHYWNYWG; L-CDR1: SCGASQSVSSNYLAWY, L-CDR2: IYDASSRATGI, L-CDR3: YCQQYGSSPLTFG). Their distribution, visualized in pearl-necklace plots, showed contact regions as densely clustered beads along Fab sequences (Figure 3). Consistently, the heavy chain paratopes spanned longer segments than those of the light chains, underscoring their dominant role in antigen recognition. This representation facilitated inter-isotype comparison and revealed conserved patterns that may underpin the broad functional versatility of immunoglobulins.

The 179 epitopes obtained from the *in silico* analysis of the *T. cruzi* proteome were modeled using PEP-FOLD 3.5, preserving structural motifs compatible with antibody binding. Their compactness and lack of disordered regions favored the subsequent docking phase. The three-dimensional prediction allowed preliminary inspection of key residue accessibility and the elimination of sterically unviable conformations, ensuring that only biologically relevant structures advanced to immunological recognition assessment *via* molecular docking.

#### 3.2.3 Molecular docking and dynamic stability analysis of the immune complex

**Figure 4:**
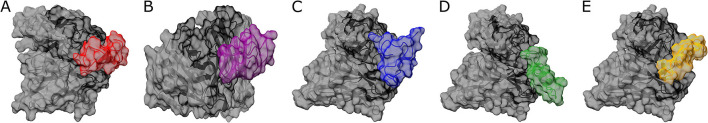
Molecular docking models depicting epitope binding to immunoglobulin FAB regions: **(A)** Epitope bound to IgA, **(B)** Epitope bound to IgM, and **(C–E)** Distinct epitopes bound to IgG. The FAB region is highlighted in gray, the paratope in black, and epitope colors **(A–E)** correspond to the sequences specified in Table 2.

Parallel docking with HPEPDOCK 2.0 and HawkDock revealed high-affinity interactions selective for each immunoglobulin class. In total, 1 epitope was identified for IgA (HPEPDOCK: −216.46; HawkDock: −3912.81), 1 for IgM (HPEPDOCK: −220.14; HawkDock: −3421.17), and 3 for IgG (Ep2 HPEPDOCK: −240.53, HawkDock: −3907.48; Ep3 HPEPDOCK: −211.97, HawkDock: −3607.84; Ep4 HPEPDOCK: −227.15, HawkDock: −4260.45), all with scores above the mean + 1 standard deviation in both tools (Table 2). The distribution of these final candidates across different isotypes (one targeting IgA, one for IgM, and three for IgG) was not pre-determined. Rather, it was the unbiased outcome of the strict independent consensus threshold applied to the docking scores, highlighting the sequences with the highest theoretical binding affinities regardless of the immunoglobulin class. Visualizations in UCSF ChimeraX confirmed consistent binding geometries: the epitopes nestled into the cavities outlined by the paratopes, forming complementary hydrogen bond networks and hydrophobic contacts (Figure 4).

**Table 2:** *T. cruzi* B-cell epitope sequences exhibiting high predicted binding affinity to human immunoglobulins.

No. Epitope	Epitope sequence	Three-dimensional representation	Immunoglobulin recognition
1	VGSDTTRYDNVNNDGGMWVKDGWD	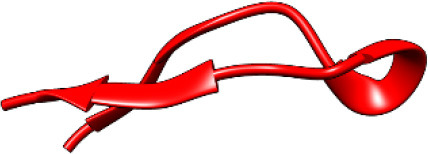	IgA
2	TLCGRDPVNPGAHLHTPPSLPRAR	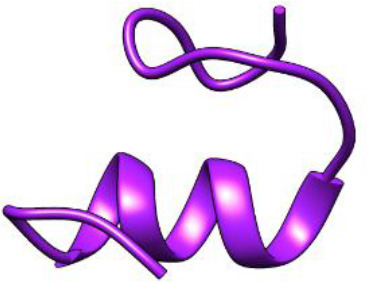	IgM
3	NGHGDDESLVREKLYCEFRT	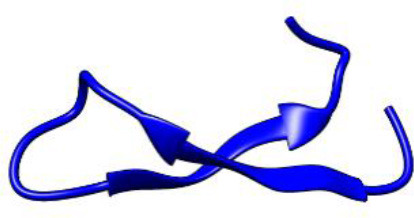	IgG
4	FVTGPPGNYRRELMYCADPENNTAR	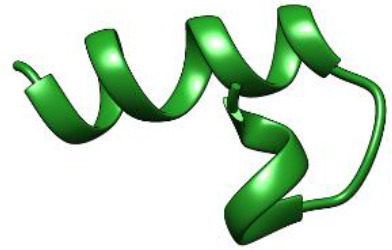	IgG
5	VEAIIHNPDPETYTYCRDKNY	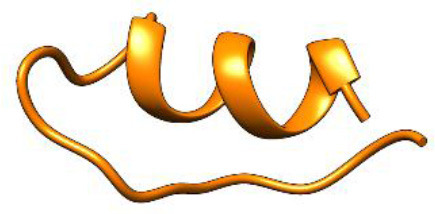	IgG

Furthermore, *in silico* physicochemical evaluation confirmed the structural viability of these five selected sequences (ranging from 20 to 25 amino acids) as robust diagnostic targets (Table 3). All candidates were classified as stable, exhibiting instability indices well below the degradation threshold (2.19–32.90) and high hydrophilicity, as denoted by negative grand average of hydropathicity (GRAVY) values (−1.200 to −0.675), alongside diverse theoretical isoelectric points (3.97–10.26) and aliphatic indices indicating thermostability (35.20–69.17). Ultimately, applying the predefined statistical threshold and integrating these optimal physicochemical profiles, the five top-scoring complexes were prioritized for subsequent molecular dynamics simulations.

**Table 3:** *In silico* physicochemical properties of selected *T. cruzi* B-cell epitopes.

No. Epitope	No. aminoacid	Theoretical pI	Instability index	Classified protein	Aliphatic index	Grand average of hydropathicity
1	24	3.97	12.05	stable	36.25	−1.200
2	24	10.26	27.12	stable	69.17	−0.675
3	20	4.90	2.19	stable	53.50	−1.180
4	25	6.17	32.90	stable	35.20	−1.012
5	21	4.75	21.90	stable	55.71	−1.124

**Figure 5:**
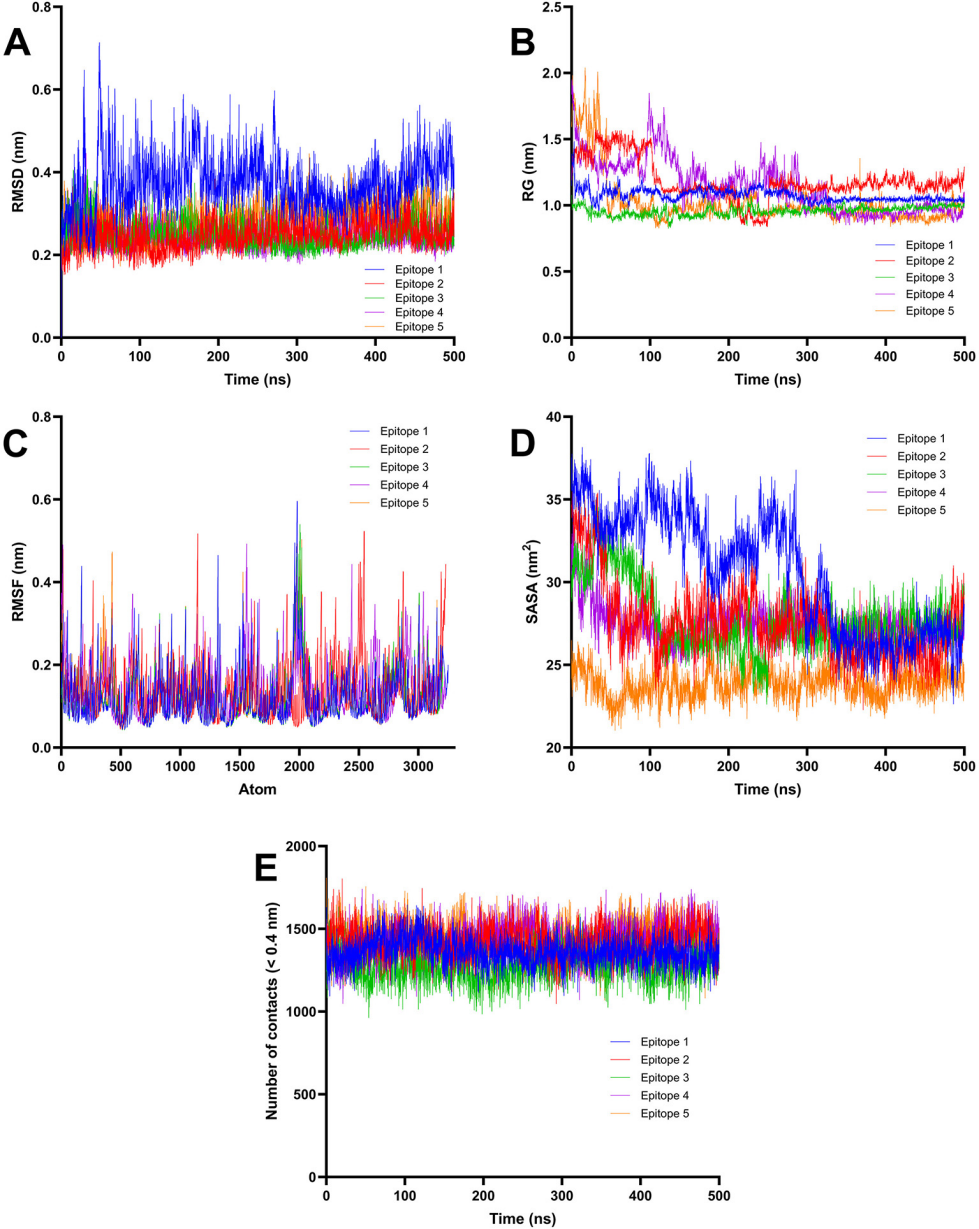
Molecular dynamics trajectory analysis (500 ns) of the antibody-epitope complexes. **(A)** RMSD of the protein backbone as a function of time. **(B)** RG of the complexes throughout the simulation, indicating the degree of structural compactness. **(C)** RMSF per atom, evidencing the residual flexibility of the protein regions. **(D)** SASA as a function of time, demonstrating changes in exposure to the aqueous environment. **(E)** Time evolution of the number of intermolecular atomic contacts formed at the binding interface at a threshold distance of < 0.4 nm. The trajectories corresponding to each complex are color-coded: Epitope 1 (blue), Epitope 2 (red), Epitope 3 (green), Epitope 4 (purple), and Epitope 5 (orange).

To evaluate the viability of the five candidate peptides in the design of diagnostic interfaces, molecular dynamics trajectories of the antibody-epitope complexes were analyzed over 500 ns. RMSD) analysis of the protein backbone (Figure 5A) revealed that complexes formed by Epitopes 1, 2, 3, and 4 reached an early conformational equilibrium, stabilizing at RMSD values between 0.2 and 0.4 nm for most of the simulation. In contrast, the Epitope 5 complex presented significantly larger and sustained deviations, reaching maximum peaks near 0.6 nm. This suggests a lower structural affinity or a continuous induced fit that prevents rigid stabilization of the interface. The compactness of the systems was evaluated using the RG (Figure 5B). The complexes with Epitopes 1, 2, and 3 demonstrated a high level of compaction, maintaining a constant average RG of approximately 1.0 nm. The Epitope 4 complex exhibited notable initial fluctuation up to 150 ns (reaching up to ~2.0 nm) before stabilizing alongside the rest of the systems. Meanwhile, Epitope 5 maintained a consistently higher RG (~1.2 nm), corroborating the tendency for lower compactness observed in the RMSD analysis. RMSF analysis per atom (Figure 5C) identified the regions of greatest thermal mobility. While the core regions of the complexes maintained minimal fluctuations (< 0.2 nm), pronounced peaks up to 0.6 nm were observed in association with Epitopes 4 and 5, indicating high intrinsic flexibility in specific loops within these structures. Epitopes 1, 2, and 3 presented more conserved and stable RMSF profiles. SASA evidenced heterogeneous solvation dynamics among the complexes (Figure 5D). Epitope 3 maintained the lowest and most stable exposure area (~22–26 nm^2^), suggesting optimal burial within the antibody binding pocket. Notably, the Epitope 1 complex exhibited a drastic reduction in SASA, dropping from ~37 nm^2^ at the beginning of the trajectory to ~27 nm^2^ after 350 ns. This indicates a late conformational adaptation process that favors solvent occlusion at the interaction interface. The interaction network at the binding interface was quantified by the number of atomic contacts at a cutoff distance of < 0.4 nm (Figure 5E). All systems demonstrated a high density of contacts, oscillating between 1000 and 1700 throughout the 500 ns timeframe, confirming the formation of stable complexes. The complexes formed by Epitopes 4 and 5 maintained the highest and most consistent averages in the number of contacts. This correlates with the successful stabilization of their interfaces, despite the early structural variations observed in the RG of Epitope 4 and 5.

### 3.3 Proof-of-concept IgG-ELISA

#### 3.3.1 Preliminary screening and selection of candidate epitopes

To bridge the *in silico* predictions with *in vitro* diagnostic utility, a preliminary serological screening was conducted using a restricted panel of well-characterized, highly reactive CD-positive sera and healthy controls. Although all five synthesized peptides exhibited optimal physicochemical profiles, initial ELISA evaluations revealed that epitopes 1, 2, and 3 displayed suboptimal immunoreactivity or elevated background noise (non-specific binding) with negative sera, limiting their discriminatory capacity. This discrepancy between computational stability and *in vitro* recognition highlights the complex nature of natural antibody repertoires and peptide presentation on solid phases. Consequently, these three candidates were excluded from large-scale validation. Epitopes 4 and 5, which demonstrated robust antigenic recognition and high signal-to-noise ratios during this preliminary phase, were advanced as the definitive candidates for comprehensive serological assessment.

#### 3.3.2 Seropositivity rates of candidate epitopes

**Figure 6:**
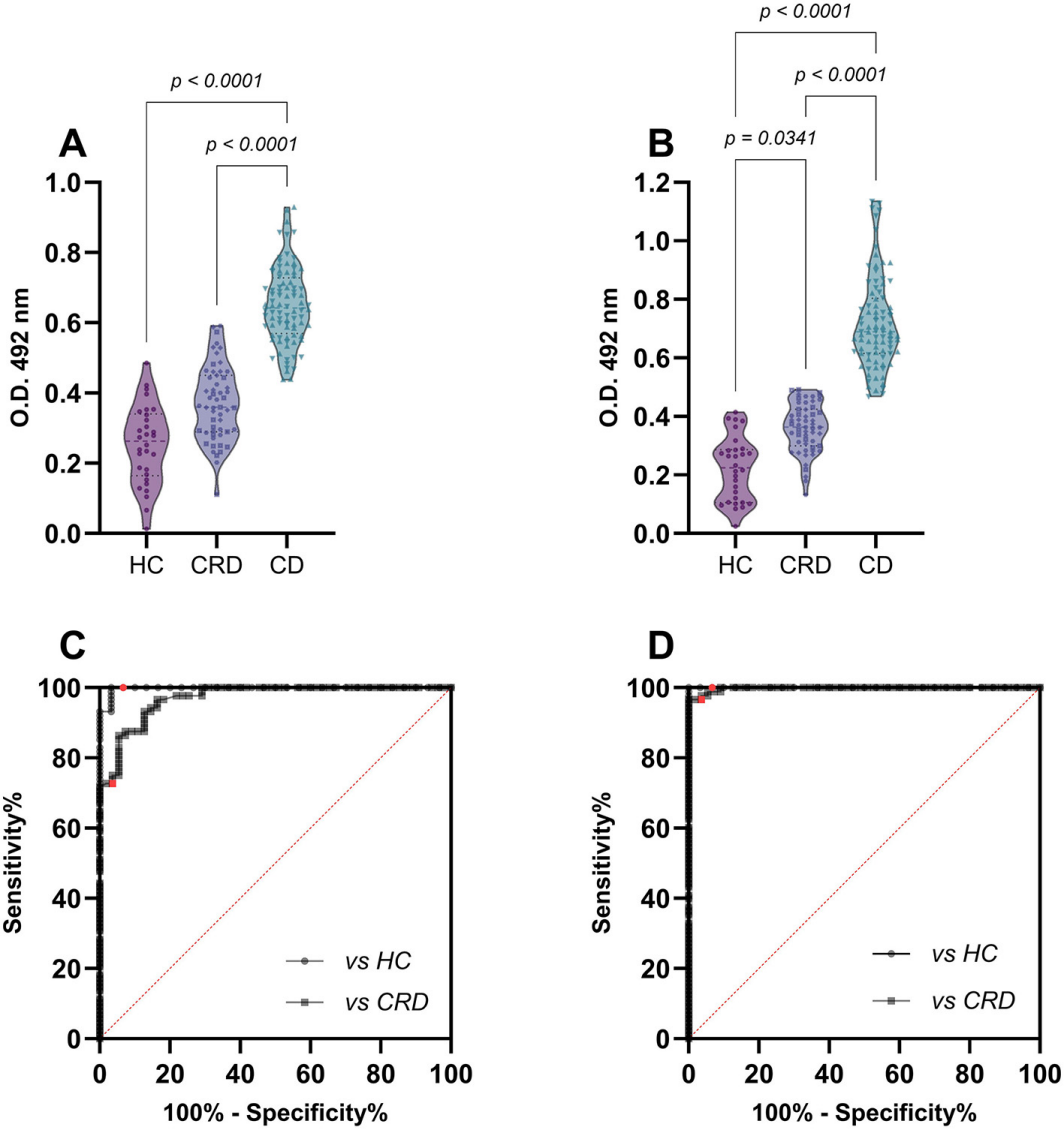
Diagnostic evaluation of epitopes 4 and 5 for Chagas disease detection. **(A, B)** Violin plots showing the optical density values at 492 nm obtained from ELISA for epitopes 4 and 5 across different groups: healthy controls (HC), cross-reactive diseases (CRD), and Chagas disease (CD). The significant differences between the groups are indicated by *p*-values. **(C, D)** Receiver operating characteristic (ROC) curves for epitopes 4 and 5, showing the sensitivity (%), specificity (%), and likelihood ratios for differentiating CD from HC and CRD.

The diagnostic potential of epitopes 4 and 5 in distinguishing Chagas disease (CD) from cross-reactive diseases (CRD) and healthy controls (HC) was evaluated using an enzyme-linked immunosorbent assay (ELISA). Sensitivity, specificity, and likelihood ratios were assessed, as summarized in Table 4 and depicted in Figure 6. Receiver Operating Characteristic (ROC) analysis demonstrated high discriminatory accuracy for epitope 4, yielding an AUC of 0.9706 (95% CI: 0.9484–0.9927, *p* < 0.0001). The diagnostic performance of epitope 4 showed 100% sensitivity across all tested groups within the evaluated cohort (CD vs. HC, CD vs. CRD), with 95% confidence intervals (CI) ranging from 95.82 to 100%. Specificity for epitope 4 was notably high, with values of 96.67% for both the CD vs. HC group. However, specificity for the CD vs. CRD group was lower at 70.91%, with a 95% CI ranging from 57.86 to 99.83%. Fisher's exact test demonstrated significant differences between the groups (*p* < 0.0001). The likelihood ratio for epitope 4 was 30.0 for the CD vs. HC group, suggesting strong diagnostic utility, especially in distinguishing CD from healthy individuals. For the CD vs. CRD group, the likelihood ratio was 3.4, indicating moderate utility in detecting cross-reactivity with other diseases. Similarly, ROC analysis for epitope 5 indicated an outstanding discriminatory capacity, with an AUC of 1.000 (95% CI: 1.000–1.000, *p* < 0.0001). Epitope 5 displayed 100% sensitivity in all tested groups within the evaluated cohort (CD vs. HC, CD vs. CRD), with 95% CI ranging from 95.82% to 100%. Specificity for epitope 5 was also 96.67% for the CD vs. HC group, with a slightly higher specificity of 90.91% for the CD vs. CRD group, and the 95% CI ranged from 80.42 to 96.05%. Fisher's exact test indicated highly significant differences across all groups (*p* < 0.0001), reinforcing the potential of epitope 5 as a reliable diagnostic marker. The likelihood ratio for epitope 5 was 30.0 in the CD vs. HC group, and 11.0 in the CD vs. CRD group, further supporting its strong diagnostic value in Chagas disease detection.

**Table 4:** Diagnostic performance evaluation of epitope against patient serum samples.

Antigens	Groups	Fisher's test	Se	95%CI	Sp	95%CI	Likelihood ratio
Epitope 4	CD x HC	< 0.0001	100	95.82–100.0	96.67	83.33–99.83	30.0
	CD x CRD	< 0.0001	100	95.82–100.0	70.91	57.86–81.23	3.4
Epitope 5	CD x HC	< 0.0001	100	95.82–100.0	96.67	83.33–99.83	30.0
	CD x CRD	< 0.0001	100	95.82–100.0	90.91	80.42–96.05	11.0

Healthy controls (HC), cross-reactive diseases (CRD), Chagas disease (CD), Sensitivity (Se), and Specificity (Sp).

## 4 Discussion

This study successfully implemented an integrated computational and experimental pipeline for the rational identification and validation of *T. cruzi*-specific linear B-cell epitopes with high diagnostic potential for Chagas disease. The comprehensive approach, spanning from proteome-wide bioinformatic screening to serological confirmation, demonstrates the feasibility of this multidisciplinary strategy and reveals critical insights into the nature of *T. cruzi*-specific immunogens and their potential to address long-standing challenges in Chagas disease diagnostics.

The initial *in silico* screening phase was essential for navigating the complexity of the *T. cruzi* proteome, which is characterized by its size, redundancy, and abundance of truncated proteins. Approximately 16% of the proteome was found to consist of truncated fragments, consistent with the fragmented state of the *T. cruzi* genome assembly and highlighting the importance of quality filtering before epitope prediction (El-Sayed et al., 2005; Reis-Cunha et al., 2018). The subsequent exclusion of proteins with significant homology to human or *Leishmania* proteomes represented a critical strategic step, distinguishing this work from earlier antigen discovery efforts. This stringent filtering, which eliminated over 4,400 proteins, aimed to minimize the risk of autoimmune reactions and cross-reactivity, the principal limitations of current serological tests based on crude lysates or purified native antigens (Afonso et al., 2012; Rodrigues et al., 2021). The resulting pool of parasite-exclusive proteins provided an ideal foundation for identifying highly specific diagnostic targets.

The computational prediction of B-cell epitopes remains challenging due to the lack of a universal gold-standard algorithm. The significant variability in the number of epitopes predicted by the five different servers (APP, BepiPred 2.0, BCPred, ABCPred, and FBCPred) reflects fundamental differences in their underlying algorithms—ranging from propensity scales and machine learning to structural propensity indicators (Potocnakova et al., 2016; Sanchez-Trincado et al., 2017). Rather than viewing this discrepancy as a limitation, the study leveraged it to implement a consensus strategy, increasingly recognized as a best practice in computational immunology (Dhanda et al., 2018; Nezafat et al., 2014). By requiring agreement across multiple prediction tools, confidence in the candidate epitopes was significantly increased. The stratification of predictions based on the level of agreement created a valuable confidence gradient, with the 401 epitopes identified by all five tools representing the highest-confidence candidates with minimal likelihood of being false positives.

An innovative aspect of the pipeline was the incorporation of structural stability assessment early in the selection process. While many *in silico* studies focus solely on linear immunogenicity, it was recognized that a synthetic peptide must maintain a stable conformation in solution to function effectively in diagnostic assays (Duvaud et al., 2021; Gasteiger et al., 2005). The application of the instability index filter eliminated over half of the top consensus epitopes, highlighting that immunogenic potential alone is insufficient for predicting diagnostic utility. This finding aligns with the growing recognition that intrinsically disordered peptides often fail to mimic native epitopes and perform poorly in serological assays (Regenmortel, 2009). Structural modeling with PEP-FOLD 3.5 provided further confirmation that the selected epitopes possessed compact, well-folded structures compatible with antibody recognition.

The structural characterization of human Fab paratopes across different isotypes revealed important insights into potential binding interactions. The observed differences in CDR profiles between IgA, IgG, and IgM isotypes corroborate established knowledge about structural variations in antibody binding sites (Mix et al., 2006), while the predominance of heavy chain interactions aligns with the well-documented central role of heavy chain CDR3 regions in antigen recognition and specificity determination (D'Angelo et al., 2018). These structural analyses provided a rational basis for expecting isotype-specific binding patterns in subsequent docking experiments.

The molecular docking analysis revealed thermodynamically favorable binding affinities for the selected epitope-isotype pairs, yielding energy scores that significantly surpassed statistical thresholds. This differential *in silico* affinity profile—identifying distinct interactions for IgA (1), IgM (1), and predominantly IgG (3)—provides a robust structural framework for interpreting the complex humoral response observed in Chagas disease. Crucially, the translational rationale linking our multi-isotype modeling to an IgG-focused experimental validation is grounded in the clinical kinetics of *T. cruzi* infection; while computational docking explored the full landscape of antibody interactions to characterize the comprehensive immunogenic potential of the epitopes, our wet-lab validation strictly prioritized IgG detection, as persistently elevated IgG titers constitute the serological gold standard for diagnosis during the chronic phase (Rassi et al., 2010). Consequently, whereas the prediction of IgM and IgA interactions maps the theoretical breadth of epitope surface accessibility, the experimental corroboration of IgG confirms practical diagnostic utility in the chronic setting, a conclusion further reinforced by the visualization of dense interaction networks—stabilized by hydrogen bonds and hydrophobic contacts—within the paratope cavities (Vajda et al., 2017).

The molecular dynamics simulations substantially strengthened confidence in these interactions by demonstrating their stability over biologically relevant timescales. The consistently low RMSD values (1.3–2.4 Å) throughout the 500 ns simulations indicated that the complexes maintained stable binding modes with minimal deviation from their initial conformation, a key indicator of biological relevance (Filipe and Loura, 2022; Hollingsworth and Dror, 2018). The preservation of secondary structure elements (%SSE) further supported the conclusion that these epitopes form stable complexes with their cognate antibodies, meeting a critical requirement for diagnostic applications where stable antigen-antibody interactions are essential for assay reliability.

The transition from *in silico* prediction to experimental validation represented a significant challenge in computational immunology, yet the successful synthesis and purification of all five candidate peptides with high yield and purity (>95%) demonstrated the practical feasibility of the approach. The serological performance of epitopes 4 and 5 was exceptional within the evaluated cohort, achieving perfect sensitivity (100%) across all patient groups. While these initial findings represent a rare achievement that surpasses most previously reported Chagas disease biomarkers, further validation in larger, multi-centric cohorts is required to establish definitive clinical sensitivity (Afonso et al., 2012; Candia-Puma et al., 2022). This performance is particularly notable when compared to commercial ELISA kits and chemiluminescent assays that rely on crude lysates or recombinant antigens, which often exhibit batch-to-batch variability and inconclusive results in “gray zone” patients (Santos et al., 2016). Crucially, the high specificity against both healthy controls (96.67%) and patients with cross-reactive diseases (90.91%) demonstrated specifically by epitope 5 addresses the longstanding challenge of distinguishing *T. cruzi* from *Leishmania* spp. in endemic regions (Caballero et al., 2007; Freitas et al., 2022). Unlike traditional biological antigens, the strictly defined chemical structure and superior thermal stability of our synthetic peptides position them as ideal candidates for lateral flow immunochromatographic assays, offering a robust alternative for Point-of-Care testing in rural areas where laboratory infrastructure is limited (Elkheir et al., 2021).

The transition from *in silico* prediction to experimental validation represented a significant challenge in computational immunology, and the successful synthesis and purification of all five candidate peptides with high yield and purity (>95%) demonstrated the practical feasibility of the approach. The serological performance of epitopes 4 and 5 was exceptional, with perfect sensitivity (100%) across all patient groups, a rare achievement that surpasses most previously reported Chagas disease biomarkers (Afonso et al., 2012; Candia-Puma et al., 2022). Even more notably, the high specificity against both healthy controls (96.67%) and patients with cross-reactive diseases (90.91%) represents a potentially transformative advance. This specificity is particularly significant given the longstanding challenge of serological cross-reactivity between *T. cruzi* and other pathogens, especially *Leishmania* spp., in endemic regions (Caballero et al., 2007; Freitas et al., 2022).

To understand the biological basis of this exceptional immunoreactivity, we examined the parent proteins of our top-performing candidates. Epitope 4 is derived from a putative glycosyl transferase-like protein (UniProt ID: Q4D163), an enzyme class intimately associated with the metabolic processes and the synthesis of surface glycoconjugates that characterize the parasite's interface with the host (El-Sayed et al., 2005). Given the annotated membrane association of this protein, it is biologically plausible that this epitope represents an accessible surface motif, facilitating the robust antibody recognition observed in our study. Conversely, Epitope 5 originates from an uncharacterized protein (UniProt ID: Q4DP55; El-Sayed et al., 2005), a finding that underscores the strategic value of our unbiased, proteome-wide screening. By successfully mining a highly diagnostic marker from the unannotated “dark matter” of the *T. cruzi* genome, this result validates the pipeline's capacity to identify novel immunogens that would be missed by traditional approaches focused solely on known, historically immunodominant antigens (Caradonna and Schmidt, 2021). Consequently, by not applying a restrictive cellular localization or surface-exposure filter *a priori*, the present methodology effectively bypasses historical biases. This unbiased strategy demonstrates that biologically relevant and highly accessible diagnostic targets can be successfully identified within the uncharacterized proteome.

The statistical measures of diagnostic performance further underscore the potential utility of these epitopes. The positive likelihood ratios of 30.0 for both epitopes 4 and 5 against healthy controls, alongside 3.4 for epitope 4 and 11.0 for epitope 5 against cross-reactive syndrome patients, indicate that positive test results based on these epitopes would generate large and conclusive shifts in pre-test probability (Deeks and Altman, 2004; Fierz and Bossuyt, 2020). These values far exceed the threshold of 10 that is typically considered indicative of a highly informative diagnostic test.

The statistical measures of diagnostic performance further underscore the potential utility of these rationally designed epitopes. The positive likelihood ratios of 30.0 for both epitopes 4 and 5 against healthy controls indicate a robust baseline specificity. Crucially, when segregating the cross-reactive cohort by specific underlying infections, the epitopes maintained high discriminatory power. Overcoming cross-reactivity with closely related trypanosomatids, particularly *Leishmania* spp., remains the primary bottleneck in Chagas disease immunodiagnostics due to extensive phylogenetic and antigenic homology (Daltro et al., 2019; Umezawa et al., 1999). By achieving sustained positive likelihood ratios (3.4 for epitope 4 and 11.0 for epitope 5) against defined cross-reactive subgroups, our findings indicate that the *in silico* selection of these epitopes successfully evades conserved pan-trypanosomatid regions. Consequently, positive test results based on these epitopes generate large and conclusive shifts in pre-test probability (Deeks and Altman, 2004; Fierz and Bossuyt, 2020), with epitope 5 exceeding the threshold of 10 typically indicative of a highly informative diagnostic test. Furthermore, the consistent recognition of these epitopes across distinct clinical forms of Chagas disease highlights their universal diagnostic coverage, regardless of the patient's clinical staging.

When contextualized within the existing literature, our findings represent a significant paradigm shift in Chagas disease diagnostics, moving from empirical antigen selection to high-throughput rational design. Historically, diagnostic efforts have relied on a restricted set of immunodominant proteins—such as Recombinant antigen B13, Cruzain Related Antigen, or Flagellar Repetitive Antigen—which, despite their utility, frequently compromise specificity due to shared epitopes with co-endemic kinetoplastids like *Leishmania* spp. and *Trypanosoma rangeli* (Bottino et al., 2013; Resende et al., 2024; Ribeiro et al., 2024); however, by implementing a proteome-wide scanning strategy augmented by stringent homology filtering and multi-tiered computational validation, we successfully circumvented these limitations to identify novel B cell epitopes phylogenetically unique to *T. cruzi* (Michel-Todó et al., 2019; Trevisan et al., 2020). These candidates not only offer the requisite stability to anchor a new generation of high-precision point-of-care assays critically needed in resource-limited settings (Suárez et al., 2022) but also enable the engineering of multi-epitope fusion proteins that enhance sensitivity without sacrificing established specificity (Dias et al., 2024; González-López et al., 2022).

Nevertheless, despite these promising results, this study presents limitations that define the roadmap for future validation. While the current sample size (*n* = 178) was sufficient for proof-of-concept analysis, rigorous assessment in larger, multi-centric cohorts is necessary. Specifically, our next phase of validation involves recruiting a cohort from the southern region of Peru (e.g., Arequipa) and the Gran Chaco region. This geographic expansion is critical to evaluate the diagnostic performance of our epitopes against different *T. cruzi* Discrete Typing Units (DTUs). Since the genetic diversity of *T. cruzi* varies significantly by geography (e.g., TcI vs. TcII/TcV/TcVI), testing sera from these distinct epidemiological scenarios will ensure that the identified epitopes are universally conserved and not limited to a specific strain circulating in the initial study population (Levy et al., 2006; Zingales et al., 2012). Furthermore, while this proof-of-concept study successfully targeted the primary diagnostic barrier of *Leishmania* spp. cross-reactivity, future studies must include sera from *T. rangeli*-infected patients, as well as samples from individuals with syphilis and autoimmune diseases, to strictly rule out other potential sources of cross-reactions. Evaluating these epitopes in acute-phase sera will also be necessary to delineate their full diagnostic utility.

## 5 Conclusions

In conclusion, this study successfully demonstrates the power of an integrated *in silico* and experimental pipeline for the rational design of highly specific diagnostic antigens for Chagas disease. By implementing a stringent bioinformatic strategy that excluded proteins with homology to *Leishmania* spp. and *H. sapiens*, followed by multi-algorithm consensus epitope prediction and physicochemical stability filtering, we identified novel *T. cruzi*-specific B-cell epitopes. The exceptional serological performance of the lead candidates, achieving 100% sensitivity and over 90% specificity within the tested cohort, even against cross-reactive diseases, validates the central hypothesis and underscores the critical importance of reducing homology to eliminate false positives. This work not only provides a robust foundation for the development of a transformative next-generation immunoassay but also establishes a reproducible blueprint for targeted epitope discovery in complex pathogens, offering a promising path toward improved epidemiological surveillance and disease management in endemic regions.

## Data Availability

The original contributions presented in the study are included in the article/supplementary material, further inquiries can be directed to the corresponding author.
